# Dengue in Patients with Central Nervous System Manifestations, Brazil

**DOI:** 10.3201/eid1804.111552

**Published:** 2012-04

**Authors:** Fernanda Araújo, Rita Nogueira, Maurício de Sousa Araújo, Anne Perdigão, Luciano Cavalcanti, Raimunda Brilhante, Marcos Rocha, Dina Feitosa Vilar, Suzana Silveira Holanda, Deborah de Melo Braga, José Sidrim

**Affiliations:** State Health Secretariat of Ceará, Fortaleza, Ceará, Brazil (F.M.C. Araújo, A.C.B. Perdigão, D.C.L. Feitosa Vilar, S.G. Silveira Holanda and D.N. de Melo Braga);; Oswaldo Cruz Institute, Rio de Janeiro, Rio de Janeiro, Brazil (R.M.R. Nogueira);; Dr. José Frota Institute, Fortaleza (M. de Sousa Araújo);; Federal University of Ceará, Fortaleza (L.P.G., Cavalcanti, F.M. de Carvalho Araújo, J.J. da Costa Sidrim, R.S.N. Brilhante, M.F.G. Rocha);; State University of Ceará, Fortaleza (A.C.B. Perdigão);; College Christus, Fortaleza (L.P.G. Cavalcanti)

**Keywords:** dengue, viruses, cerebrospinal fluid, viral meningitis/meningoencephalitis, Brazil

## Abstract

We investigated the prevalence of dengue in patients with suspected viral meningitis/meningoencephalitis in a dengue-endemic area. Cerebrospinal fluid analysis showed positive results and a 6.74× greater likelihood of identifying positive fluid in patients who died. Our findings support testing patients with neurologic manifestations for the virus in dengue-endemic areas.

Dengue is the most prevalent arboviral infection in humans (*1*). Since the reintroduction of dengue virus (DENV) into Brazil in the 1980s, >60% of the reported dengue cases in this region of the Western Hemisphere have occurred there (*2*). As the disease has become more common, unusual clinical signs, some of which involve the central nervous system, have been observed in dengue patients (*2*–*4*). We therefore assessed prevalence of dengue neurologic cases from Ceará State, Brazil, a region where dengue is endemic.

## The Study

We enrolled 183 patients with suspected viral meningitis/meningoencephalitis admitted to São José Hospital of Infectious Disease and 26 deceased patients with suspected fatal meningitis who had been sent to the city of Fortaleza Coroner’s Office. Cerebrospinal fluid (CSF) was collected from all 209 patients. Study inclusion criteria were suspicion of viral meningitis/meningoencephalitis, a CSF cell count <500 cells/mm^3^, and negative results of culture and microscopic examination for bacteria and fungi. The CSF samples were not contaminated with blood. The study was performed retrospectively and used samples from patients who had been treated for meningitis during 2005–2008, a period during which a dengue epidemic may have occurred in Ceará. This study was approved by the Ethics Committee of São José Hospital of Infectious Disease (protocol no. 005/2009; Certificado de Apresentação para Apreciação Ética [Proof of Application for Ethical Review] 0005.0.042.000–09).

Dengue meningitis was suspected when a patient had fever and symptoms of irritation of the meninges, such as headache and neck stiffness; a diagnosis of dengue meningoencephalitis was established when the patient showed signs of focal involvement of the central nervous system (CNS). A diagnosis of dengue was confirmed with a DENV-positive CSF result by reverse transcription PCR (RT-PCR), nonstructural protein (NS) 1, or IgM against DENV (*3*,*4*).

Samples were analyzed by using RT-PCR, ELISA for NS1, and IgM monoclonal antibody and a rapid immunochromatography test for IgG (*3*–*5*).Viral RNA for the nested RT-PCR was extracted from 140 µL of the CSF samples by using the QIAamp Viral RNA Mini Kit (QIAGEN, Valencia, CA, USA), following the manufacturer’s protocol, and stored at −80°C until tested. The RT-PCR for DENV was performed on 209 CSF samples, as described (*5*).

The NS1Ag Pan-E Dengue Early ELISA kit (Panbio Diagnostics, Brisbane, Queensland, Australia) was used to detect the dengue NS1 in 209 CSF specimens in accordance with the manufacturers’ instructions (*4*). The Dengue IgM Capture ELISA (Panbio Diagnostics) was performed on 209 CSF samples, according to the manufacturer’s instructions. The Panbio Dengue Duo Cassette rapid test was performed, according to the manufacturer’s instructions, with CSF specimens that were positive for DENV in any of the other tests used.

Of 209 CSF samples studied, 8 (3.8%) showed positive results in >1 test: 5 from the group admitted to São José Hospital of Infectious Disease and 3 deceased patients examined at the Fortaleza Coroner’s Office (Table 1). Reviewed literature showed that the etiologic agents of most cases of viral meningitis in Brazil are enterovirus and herpesvirus; cytomegalovirus and dengue viruses are each responsible for 10% (2/20) (*6*).

**Table 1:** Clinical features and virologic findings for 8 patients with meningitis/meningoencephalitis and confirmed cases of dengue, Brazil, 2005–2008*

Patient no.	Age, y/sex	Initial symptoms and signs	Progress and outcome	RT-PCR	NS1Ag	IgM	IgG	ND
1	45/M	Fever, headache, sweating, thorax pain, seizure, coma, chronic hypertension.	Cerebral edema and congestion; mononuclear cells in meninges; death after 6 d	–	+	–	–	ME
2	32/F	Fever, vomiting, neck stiffness, myalgia, abdominal pain, asthenia, somnolence, confusion	Meningitis, sixth nerve palsy; death after 14 d	DENV-3	+	–	–	ME
3	1/M	Fever, tremors, rigidity of limbs, otitis	Intracranial hypertension, meningitis; death after 24 h	–	+	+	–	M
4	6/F	Fever, headache, malaise, vomiting, drowsiness, neck stiffness	CSF: clear, 133 cells/mm^3^, 42% lymphocytes, 2% monocytes, 53% neutrophils, 3% eosinophils; protein 58 g/L, glucose 54 g/L; recovery after 9 d	–	–	+	+	M
5	58/M	Fever, headache, severe malaise, vomiting, lowering of consciousness, delirium	CSF: 300 cells/mm^3^; lymphocytes, 87%, monocytes 5%, neutrophils 4%, protein 112 g/L, glucose 59 g/L; serum: AST 127 U/L, ALT 74 U/L; CT scan: expansible lesion measuring 4 × 2 x 2.3 cm; referred for surgical treatment	–	–	+	+	Brain tumor; M
6	5/F	Fever, headache, vomiting, neck stiffness	CSF: 490 cells/mm^3^, 2% monocytes, 5% lymphocytes, 93% neutrophils, protein 45 g/L, glucose 110 g/L; recovery after 8 d	–	–	+		M
7	15/M	Fever, headache, arthralgia, severe malaise, dry cough, dyspnea, epigastric pain	IHC result positive for dengue. CSF: clear; cerebrum and cerebellum with marked edema and vasocongestion of meninges and nerve tissue; death after 5 d	–	+	–	–	ME
8	24/M	Fever, headache, vomiting, and neck stiffness	CSF: 426 cells/mm^3^; protein 136 g/L, glucose 55 g/L; recovery	–	–	+	+	M

*RT-PCR, reverse transcription PCR; NS1Ag, nonstructural protein 1 antigen; ND, neurologic diagnosis; –, negative; +, positive; ME, meningoencephalitis; DENV, dengue virus; M, meningitis; CSF, cerebrospinal fluid; AST, aspartate aminotransferase; ALT, alanine aminotransferase; CT, computed tomography; IHC, immunohistochemical test.

## Conclusions

DENV as a causal agent for meningitis has been rarely reported, although some cases have been described in the literature. In Jamaica, a study of 401 patients with suspected cases of viral infection of the CNS showed that 54 (13.5%) were positive for dengue; 18 (33.3%) of those patients showed clinical signs of meningitis (*7*). However, when we included patients in the cohort who were initially suspected of having CNS infection, the frequency of meningitis in this study was 18/401 (4.5%).

An investigation of dengue patients with suspected CNS infection conducted in Vietnam found 4.2% (16/378) of persons positive for DENV; 1 (0.3%) patient had meningitis (*3*). The frequency of finding dengue virus in patients with suspected cases of meningitis found in this study corroborated what was hypothesized in the literature: neurologic manifestations in patients with DENV have been reported in Ceará, but previous studies based laboratory diagnosis on serum, not on CSF as in our study, which indicated a relationship between dengue and CNS manifestations (*8*).

Of 5 patients treated at São José Hospital of Infectious Disease (Table 1), 3 recovered, 1 was given a diagnosis of a brain tumor, and 1 died. The patient who died was the only person of 5 with dengue fever who had signs and symptoms of fatal dengue hemorrhagic fever (DHF) (such as intense malaise, dry cough with dyspnea, and abdominal pain) (*9*). Of the 3 deceased patients (Table 1), only 1 had signs of severe dengue, including myalgia, abdominal pain, asthenia, somnolence, and confusion. Suspected cases of meningitis with other pathologic changes might also be confused with dengue cases with CNS involvement (*10*). Of 8 dengue patients, 2 had signs and symptoms of dengue infection. In Brazil, meningitis was confirmed for patients with oligosymptomatic dengue infection in the cities of Vitoria and Rio de Janeiro (*10*,*11*).

The presence of DENV NS1 antigen (NS1Ag) has been associated with virus replication and viremia with the risk for development of DHF (*12*). The NS1Ag was detected in 4 of the fatal cases reported here, but because none fulfilled the World Health Organization criteria for DHF, they were considered to have been cases of severe dengue because the patients died (*1*) (Table 1). Detection of dengue IgM in CSF has shown a high specificity (97%) for diagnosing neurologic dengue and might be associated with the neurovirulence of DENV and its ability to cause encephalitis (*13*). Prior to the 1996 publication of findings by Lum et al., involvement of the CNS in dengue infection had been thought to be secondary to vasculitis only; direct involvement of the brain by DENV was thought to be unlikely (*14*),. The literature has reported detection of DENV in the brain and CSF by PCR and virus isolation and detection of NS1 and dengue IgM, providing strong evidence that DENV has neurovirulent properties (*3*,*4*,*11*,*13*–*15*). Meningeal lesions, neuronal damage, and evidence of DENV in CSF by RT-PCR and ELISA (NS1/IgM) found in this study are consistent with CNS infection (Table 1).

The prevalence of CNS involvement in patients with dengue infection seems to vary with severity of dengue cases (*11*). Mortality rates also vary among studies; the reported rate of neurologic dengue was found to be 3.7% (2/54) in a study in Jamaica (*7*). In another study conducted in Vietnam, no patients with the neurologic form of dengue died (*3*); our study found a mortality rate of 1.9% (4/209). However, the proportional positivity was higher for the group of patients who died (4/27, 14.8%) than for those who recovered (4/182, 2.2%) (Table 2). The relative risk for identifying DENV-positive CSF in patients who died was 6.74× greater than that for patients who recovered (95% CI 1.79×–25.38×; p<0.0109). No patients had DHF or a concurrent condition to predict deterioration to death, thus suggesting that patients with meningitis/meningoencephalitis and DENV-positive CSF may have higher risk for development of severe forms of dengue infection.

**Table 2:** Risk for death among patients with meningitis/meningoencephalitis with DENV+ versus DENV– cerebrospinal fluid test results, Brazil, 2005–2008†

Outcome	DENV+	DENV–	Total
Death	4 (14.8)	23 (85.2)	27 (100)
Recovery	4 (2.2)	178 (97.8)	182 (100)

*Values are no. (%) patients. Relative risk 6.74 (95% CI 1.79–25.38); p<0.0109. DENV, dengue virus; –, negative; +, positive.

The high risk for death among patients with dengue meningitis/meningoencephalitis in this study supports the need for increased surveillance. Dengue should be suspected in patients with neurologic manifestations in dengue-endemic areas, and appropriate treatment should be given to prevent death.
